# Shape anisotropy-governed locomotion of surface microrollers on vessel-like microtopographies against physiological flows

**DOI:** 10.1073/pnas.2022090118

**Published:** 2021-03-22

**Authors:** Ugur Bozuyuk, Yunus Alapan, Amirreza Aghakhani, Muhammad Yunusa, Metin Sitti

**Affiliations:** ^a^Physical Intelligence Department, Max Planck Institute for Intelligent Systems, 70569 Stuttgart, Germany;; ^b^Institute for Biomedical Engineering, Eidgenössische Technische Hochschule Zurich, 8092 Zurich, Switzerland;; ^c^School of Medicine, Koç University, 34450 Istanbul, Turkey;; ^d^College of Engineering, Koç University, 34450 Istanbul, Turkey

**Keywords:** medical microrobotics, surface rollers, circulatory system, vessel microtopography, microfluidics

## Abstract

Surface microrollers are promising microrobotic systems for controlled navigation in the circulatory system thanks to their fast speeds and decreased flow velocities at the vessel walls. While surface propulsion on the vessel walls helps minimize the effect of strong fluidic forces, three-dimensional (3D) surface microtopography, comparable to the size scale of a microrobot, due to cellular morphology and organization emerges as a major challenge. Here, we show that microroller shape anisotropy determines the surface locomotion capability of microrollers on vessel-like 3D surface microtopographies against physiological flow conditions. The isotropic (single, 8.5 µm diameter spherical particle) and anisotropic (doublet, two 4 µm diameter spherical particle chain) magnetic microrollers generated similar translational velocities on flat surfaces, whereas the isotropic microrollers failed to translate on most of the 3D-printed vessel-like microtopographies. The computational fluid dynamics analyses revealed larger flow fields generated around isotropic microrollers causing larger resistive forces near the microtopographies, in comparison to anisotropic microrollers, and impairing their translation. The superior surface-rolling capability of the anisotropic doublet microrollers on microtopographical surfaces against the fluid flow was further validated in a vessel-on-a-chip system mimicking microvasculature. The findings reported here establish the design principles of surface microrollers for robust locomotion on vessel walls against physiological flows.

## Significance

Controlled microrobotic navigation in blood vessels holds significant potential to revolutionize targeted drug delivery. Navigation on the surface of the blood vessels is advantageous because of decreased flow velocities; however, surface microtopography of blood vessels, in the size scale of the microrobots, is a major hurdle for robust locomotion of surface-rolling microrobots against the blood flow. Here, we show the effect of the body-shape anisotropy of the microrollers on their locomotion capability over vessel-like microtopographies. We demonstrate by experiments and simulations that the microrollers with slender bodies are more robust to locomote on biological microtopographies due to their favorable hydrodynamic interactions. Thus, such anisotropically shaped microrollers would be more viable and robust in future in vivo medical applications.

Untethered cell-sized microrobots have emerged as a promising technology for next-generation biomedical applications, especially for minimally invasive cargo delivery at hard-to-reach regions inside the human body (1–5). Although the circulatory system appears as the ideal route for reaching all organs and tissues, harsh fluidic conditions in the blood vessels impair the motion of the swimming microrobots (6–8). On the other hand, surface-rolling magnetic microrobots inspired by leukocytes can evade the strong fluidic forces by taking advantage of decreased flow velocities at the vessel walls (9, 10). While surface-rolling microrobots can evade resistive fluidic effects, heterogeneous surface topography of vessel walls poses a crucial barrier for surface locomotion. The inner layer of the blood vessels, endothelium, is covered with elongated and packed endothelial cells that possess undulating topographic features in the size scale of a few microns (11). Topographical features on the endothelium mainly stem from the following: 1) topography of individual endothelial cells due to relatively rigid nucleus with a height of ∼2 to 5 µm (11, 12); 2) organization of endothelial cells in a monolayer depending on the biophysical and biochemical conditions, such as flow profile (13–15); and 3) abnormalities within the endothelium, such as protruding cells, due to abnormal physical conditions, including disturbed blood flow (16, 17) and atherosclerosis (18). Such topographic features could deteriorate and even completely stop the translational motion of rolling microrobots due to counterintuitive hydrodynamic effects. In our previous work (9), we observed nonmonotonic motion of spherical microrollers on endothelial cell layers against the physiological blood flow. Even though microrobot rolling over large obstacles (7, 19) and on periodic sharp ridges (20) was reported previously, the effects of physiological microtopographies and flow conditions found in blood vessels on the propulsion of cell-sized microrobots remain to be investigated.

**Fig. 1.**
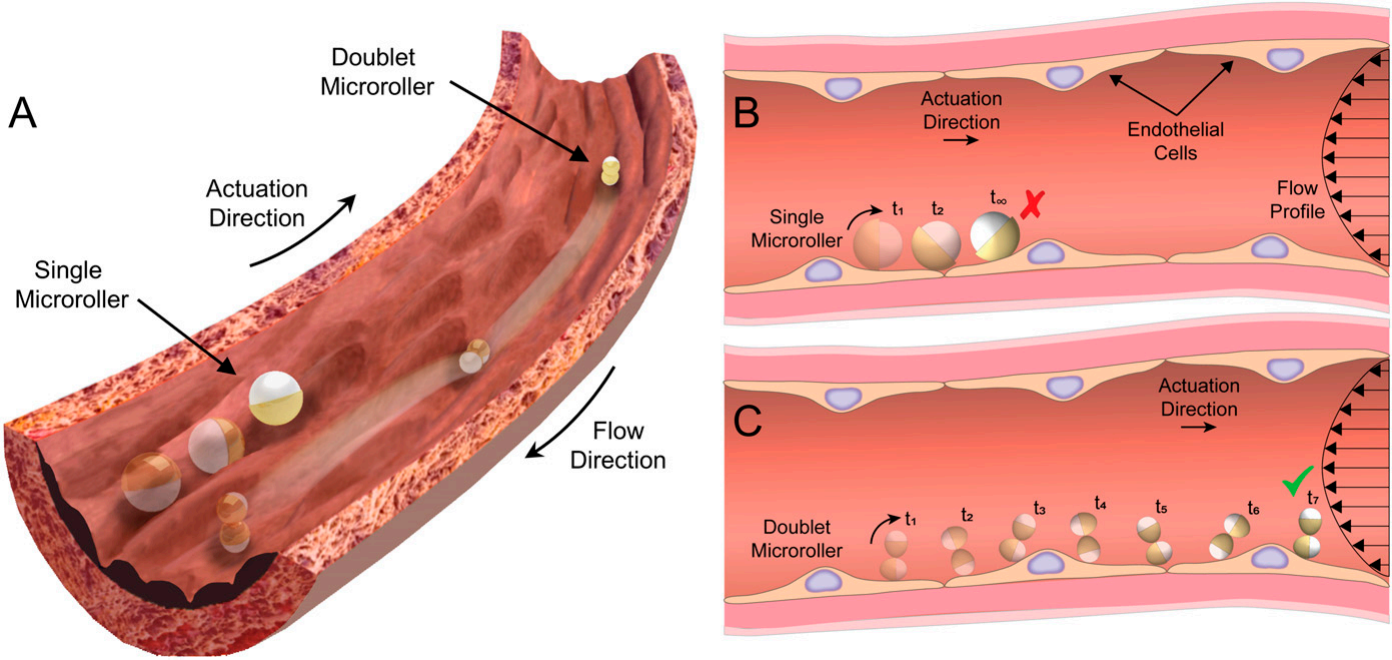
Conceptual schematic illustration of the upstream motion capability of two different (single and doublet) microrollers on endothelium in a blood vessel. (*A*–*C*) The spherical (isotropic) microrollers are prone to fail on topographical features of the endothelial cells, whereas the doublet (anisotropic) microrollers could smoothly translate.

Here, we report the effects of vessel-like surface microtopographies on the propulsion of rolling microrobots with different shapes, under physiologically relevant flow conditions. Magnetically rotated single (isotropic) and doublet (anisotropic) microrollers are composed of a single 8.5 µm diameter and 2×4 µm diameter Janus microparticles, respectively, ensuring that both have similar height but different aspect ratio. Propulsion characteristics of both groups were similar on flat glass surfaces in static and flow conditions. While the doublet microrollers could translate over most of the three-dimensional (3D) microprinted topographical surfaces emulating the vascular surface topographies in static and dynamic flow conditions, the motion of single microrollers was almost completely impaired in both cases. The two-dimensional (2D) computational fluid dynamic (CFD) analyses revealed larger flow fields generated by the single microrollers in comparison to the doublet microrollers, causing resistive forces nearby microtopographical structures against the translation direction. We further investigated the motion of the microrollers on an endothelialized microfluidic “vessel-on-a-chip” system, in which the doublet microrollers outperformed the single microrollers. The experimental findings along with the numerical simulation results suggest the employment of rolling microrobots with an anisotropic shape, such as the doublet microrollers shown in Fig. 1, for efficient and robust surface locomotion against the flow within the circulatory system.

## Results

### Propulsion of Single and Doublet Magnetic Microrollers on Flat Surfaces.

**Fig. 2.**
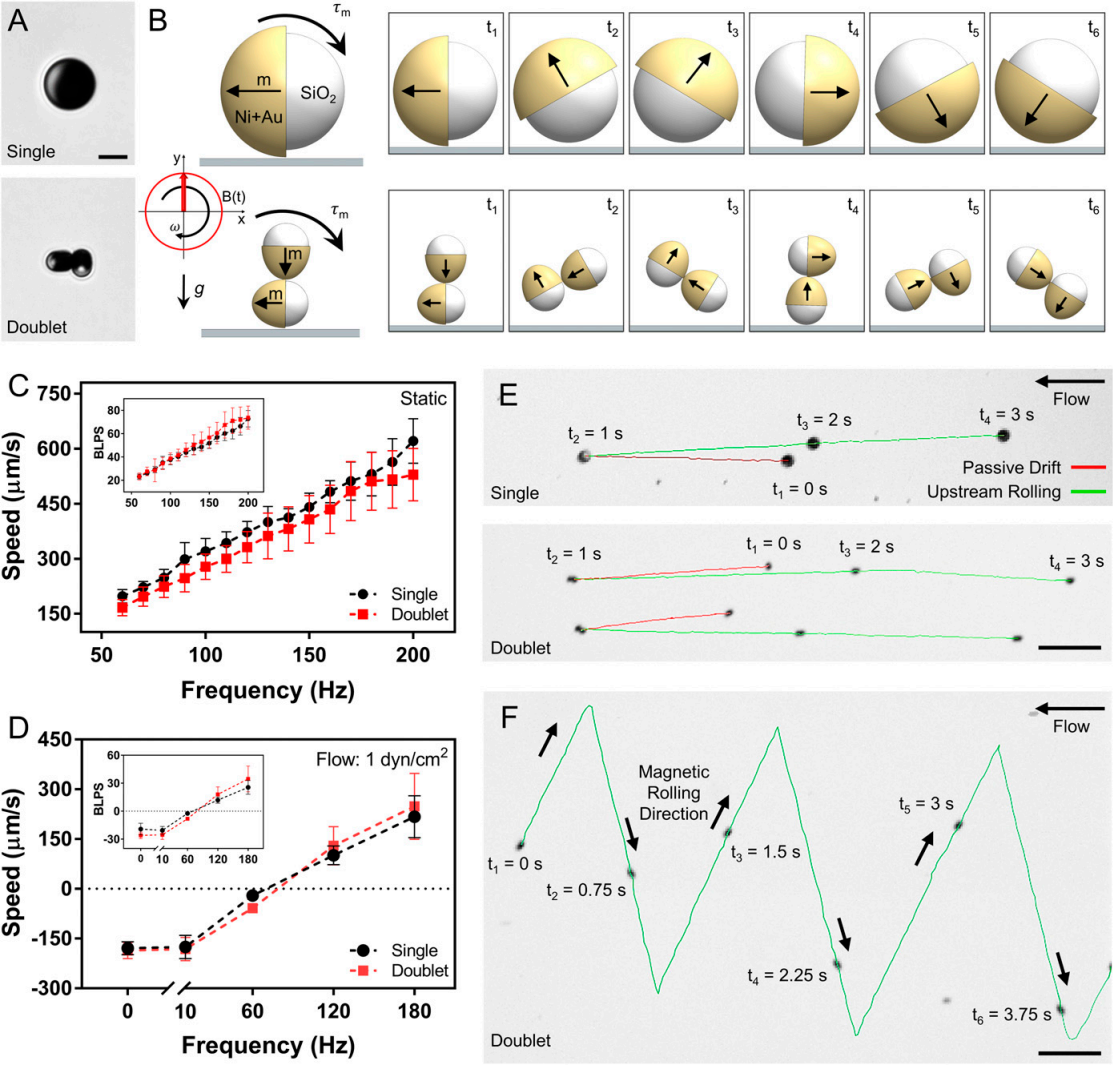
Rolling characteristics of magnetically actuated single and doublet microrollers on flat glass surfaces in a stagnant PBS (1×). (*A*) Optical microscopy images of single and doublet microrollers. Single microrollers are composed of 7.8 µm diameter silica microparticles sputtered with 1,000 nm thick Ni and 20 nm thick Au nanofilms. Doublet microrollers are composed of magnetically self-assembled 2 × 4 µm diameter silica microparticles each sputtered with 1,000 nm thick Ni and 20 nm thick Au nanofilms. (Scale bar, 5 µm.) (*B*) Under a rotating magnetic field, microrollers experience a torque (τ_m_), which results in rotation of the microrollers and hence propulsion. Black arrows show the magnetization direction of the Ni film. *g* indicates the gravity direction. (*C*) Average speed of microrollers on glass depending on actuation frequency (10 mT). Single and doublet microrollers can reach speeds up to 600 and 550 µm/s, respectively. Both groups are similar in terms of BLPS (*Inset*). Error bars represent the SD of the mean. (*D*) Frequency-dependent upstream propulsion speed of microrollers in PBS flow at 1 dyn/cm^2^. Below 60 Hz, both microroller designs fail in upstream propulsion and start drifting in the flow direction. Error bars represent the SD of the mean. (*E*) Time-lapse images of the microrollers first drifting in the flow direction (red line, until t_2_ = 1 s) and then rolling (at 180 Hz, 10 mT) against the flow (green line, until t_4_ = 3 s) at 1 dyn/cm^2^. (Scale bar, 50 μm.) (*F*) Controlled trajectory of a doublet microroller in 1 dyn/cm^2^ flow. (Scale bar, 50 μm.)

A single microroller consists of a single 7.8 µm diameter spherical Janus particle made from a silica particle with its surface half-coated with a 1,000 nm thick Ni and 20 nm thick Au film. A doublet microroller is composed of two 3 µm diameter Janus microparticles, such that each silica particle has a half surface coated with a 1,000 nm thick Ni and 20 nm thick Au film (Fig. 2*A*). The average size of the single and doublet microrollers are 8.5 and 7.2 µm, respectively, including their Ni/Au magnetic-coating layer. The rotation axis and the self-assembly configuration of the microrollers are dependent on the magnetization direction of the magnetic films, which were programmed to be out-of-plane direction. Such magnetization direction was induced on the Ni layer by applying a 1.8 T uniform magnetic field on the fabricated Janus microparticles inside a vibrating-sample magnetometer (VSM). The doublet microrollers were fabricated via magnetic self-assembly of around 4 µm diameter Janus microparticles by mixing the particle solution in relatively low concentrations (4×10^5^ particle/mL). After shaking, the particles irreversibly assembled because of strong magnetic attractions, in which around 25% of the total number of particles was self-assembled into the doublet form (*SI Appendix*, Fig. S1). All doublet experiments were performed without any further dilution/filtering, and only doublet microrollers were recorded and analyzed for further characterization. Such a magnetic self-assembly process for the doublet microrollers allowed a facile and high-throughput fabrication. The magnetic microrollers on a surface immersed in media were actuated by applying a rotating magnetic field, in which the out-of-plane–magnetized magnetic cap followed the rotating external uniform magnetic field by rolling (Fig. 2*B*). Rotating microrollers translated because of the flow field symmetry breaking with a nearby wall/surface (21–28) (Movie S1). On flat glass substrates in static conditions, single and doublet microrollers reached 600 and 550 µm/s average translational velocities, respectively, corresponding to around 72 body lengths per second (BLPS) for both designs (Fig. 2*C*). The BLPS of both designs were consistent with their length, which indicates that the microroller length is the main determinant factor for their translational speeds on flat substrates. At 10 mT rotational magnetic field magnitudes, both microroller designs were not stepped out until 200 Hz rotation frequency, which was the limit of our magnetic actuation system. In physiologically relevant flow conditions [1 dyn/cm^2^ wall shear stress in the postcapillary and small venules (29–31)] on flat surfaces, both microroller designs were able to locomote against the flow at 120 and 180 Hz but failed under 60 Hz (Fig. 2 *D* and *E* and Movie S1). Furthermore, the microrollers generated more than sufficient propulsion force for controlled upstream locomotion and navigation in 1 dyn/cm^2^ flow conditions (Fig. 2*F* and Movie S1).

### Surface Motion of Microrollers on Vessel-like Microtopographical Structures in Static Conditions.

**Fig. 3.**
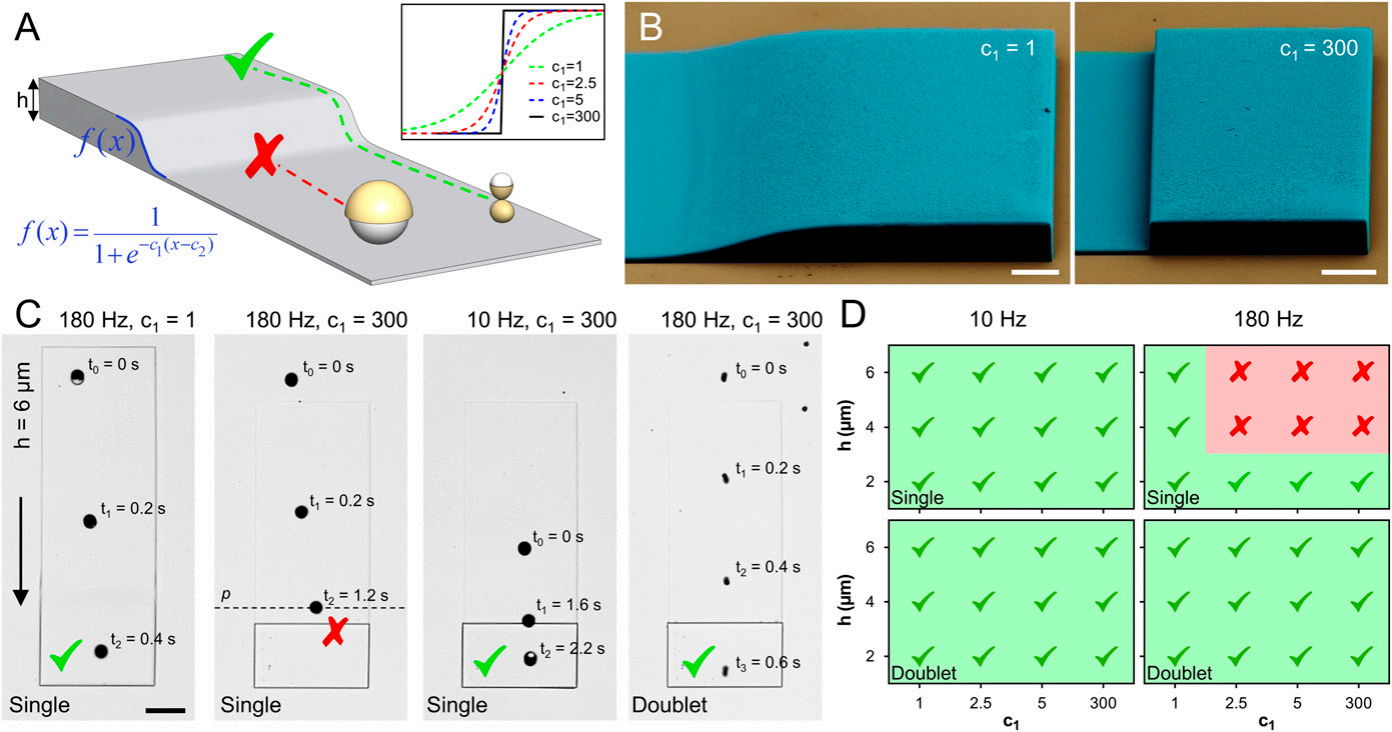
Propulsion of microrollers on microtopographical surfaces in static fluid conditions. (*A*) The microrollers are driven toward and over microfabricated topographical structures with constant profile in depth. (*Inset*) Slopes of the structures were designed according to basic sigmoid function. Microrollers that could overcome the structures were regarded as successful. (*B*) Pseudocolored SEM images of the microfabricated topographical structures. (Scale bars, 10 µm.) (*C*) Time-lapse images of the microroller propulsion on microfabricated structures (*h* = 6 µm). Single microrollers actuated at 180 Hz were able to locomote over structures with the mildest slope (*c*_1_ = 1) at high frequencies but failed at steep structures (*c*_1_ = 300). However, when actuated at 10 Hz, the same microrollers were able to climb the steep structures (*c*_1_ = 300). On the other hand, doublet microrollers propelled over all the structures at high frequencies (180 Hz). The black arrow shows the locomotion direction. The *p* denotes the stable point at which the microroller is spinning without any translation. (Scale bar, 25 µm.) (*D*) Performance diagrams of microrollers on structures with varying slopes and heights show that the doublet microrollers can move over all the structures at both low and high actuation frequencies.

To test the performance of single and doublet microrollers on topographical surfaces, we microfabricated various microtopographical structures, with a constant cross-sectional profile in depth (Fig. 3*A*), from IP-S photoresist (Nanoscribe GmbH) using two-photon lithography. We used basic sigmoid function to control the slope of the walls, where the parameter *c*_1_ adjusted the slope (Fig. 3*A*). We selected 2, 4, and 6 µm wall heights, to represent microtopographies of endothelium found in blood vessels, with different slopes (*c*_1_ = 1 to 300). Scanning electron microscopy (SEM) and confocal laser-scanning microscopy analyses showed high-fidelity 3D microprinting of the designed structures (Fig. 3*B* and *SI Appendix*, Fig. S2).

Single microrollers actuated at 180 Hz rotation frequency were able to propel over the structures with mild slopes but failed at steeper structures with higher wall heights. For example, a single microroller at 180 Hz was able to locomote over a structure with *h* = 6 µm and *c*_1_ = 1, while it failed to climb a structure with the same height but with a steeper slope (*c*_1_ = 300) (Fig. 3*C* and Movie S2). The single microroller could not fully approach the structure and was not able to move further despite constant rotational motion (Movie S2). When the same microroller was actuated at 10 Hz, it propelled over the same structure. On the other hand, the doublet microroller locomoted over all the structures, irrespective of their slope and height, at 180 Hz (Fig. 3 *C* and *D* and Movie S2). To investigate the performance limits of the doublet microroller, we performed additional experiments using the steepest slope *c*_1_ = 300 with higher wall heights (*h* = 8, 10, 15 and 20 µm). The doublet microroller failed at and above *h* = 10 µm at 180 Hz; however, it was able to locomote over the structures at 10 Hz, except for *h* = 20 µm (*SI Appendix*, Fig. S3). Overall, our experimental investigations showed that the doublet microrollers can efficiently and robustly translate over vessel-like microtopographies even at high actuation frequencies, which is required for upstream propulsion in flow conditions.

### Hydrodynamic Interactions of the Microrollers with Surrounding Structures.

**Fig. 4.**
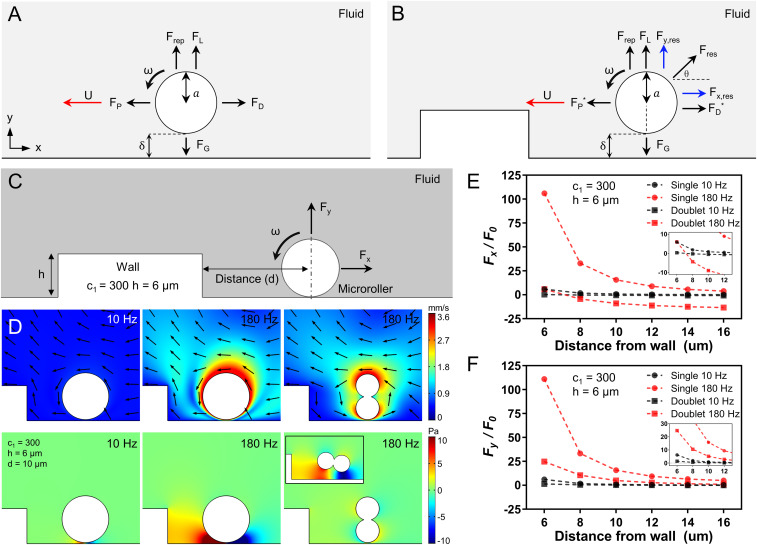
CFD analyses of the microrollers near topographical structures. (*A*) The force balance of a rolling sphere rotating on a nearby flat surface. Propulsion force (*F*_*P*_) is balanced out by the drag force (*F*_*D*_) on *x* axis, and gravitational forces (*F*_*G*_) are balanced out by repulsion (*F*_*rep*_) and lift forces (*F*_*L*_) on *y* axis. *δ* shows the separation (lubrication) distance. (*B*) In the presence of a topography, new resistive forces (*F*_*res*_) arise on both axes and affect microroller locomotion. (*C*) The 2D CFD analysis of the microrollers. A microroller with a fixed position actuated at different frequencies was gradually approached to a structure (*c*_1_ = 300, *h* = 6 µm), and total forces acting on the rollers were investigated. (*D*) Flow fields and pressure generated around the microrollers placed at 10 µm distance to the structure at different frequencies. Single microrollers actuated at 180 Hz generated larger flow fields and pressure near the structures compared to single microrollers actuated at 10 Hz and doublet microrollers actuated at 180 Hz. Colors bars indicate flow velocity and pressure. Black arrows indicate fluid flow direction. (*E* and *F*) The total forces, normalized to a constant *F*_*0*_, acting on the single microrollers at 180 Hz on both *x* and *y* axes are greater than the other groups, when near the topography.

In the presence of a nearby underlying solid wall, the rotational motion of a spherical microroller is converted into translational motion by breaking the flow field symmetry. The basic force balance on a rotating microroller in the Stokes regime is given in Fig. 4*A*. During the steady-state motion, the spherical microroller creates a propulsion force (*F*_*P*_) in its translation direction, which is balanced out by a fluidic drag force (*F*_*D*_) (32). By balancing these forces, translational velocity (*U*) can be expressed as the following:

$$F_{P}=\pi \mu a^{2}\omega f_{1}\left(\delta ,a\right),$$ [1]

$$F_{D}=6\pi \mu aUf_{2}\left(\delta ,a\right),$$ [2]

$$U=\;\frac{a\omega f_{1}\left(\delta ,a\right)}{6f_{2}\left(\delta ,a\right)},$$ [3]

where *μ* is the dynamic viscosity of the fluid, *a* is the particle radius, *ω* is angular velocity, $f_{1}$ and $f_{2}$are wall correction factors, and *δ* is the lubrication (separation) distance from the nearby wall. For a given angular velocity and microroller dimensions, translational velocity is the function of the lubrication distance which depends on the gravitational forces against buoyancy (*F*_*G*_), electrostatic repulsion (*F*_*rep*_), and lift forces (*F*_*L*_) on *y* axis (33–35), where *F*_*L*_ is negligible in the given size scale (23) (see *SI Appendix*, Note S1 for details). The increase of δ leads to the decreased translational velocity of the microrollers (32). Note that additional forces on positive *x* (against propulsion direction) and *y* axes would result in additional drag and increased lubrication distance, respectively, thus decreasing the translational speed. When a microroller approaches a microtopography, it experiences additional resistive forces (*F*_*res*_) due to extra hydrodynamic wall effects. We hypothesized that such resistive forces would lead to a new force balance (Fig. 4*B*) for the translational velocity of the microroller so that

$$U=\;\frac{a\omega {f_{1}}^{*}-F_{x,res}}{6{f_{2}}^{*}}.$$ [4]

The new translational velocity *U* includes new correction factors $\left({f_{1}}^{*},\;{f_{2}}^{*}\right)$ and *F*_*x,res*_, which depends on multiple factors, such as *δ*, *a*, *ω*, and *d*. However, exact determination of these correction factors for each unique microtopographical structure requires detailed investigation, which is beyond the scope of this study. Nevertheless, the increase in *F*_*x,res*_ and *F*_*y,res*_ would simultaneously lead to a decrease in the translational speed of the microroller, and could even stop its translation, which we have observed in our experiments (e.g., *h* = 6 μm, *c*_1_ = 300, 180 Hz). Note that, in such cases, the microroller still spins at the given magnetic rotational frequency without any translation (Fig. 3*C*, stable point).

When the force balance near the topography does not bring the microroller translation to a complete halt, the microroller can get closer to the microstructure. Within a certain distance from the topography (<1 µm) (9), the microroller’s symmetry breaking axis shifts from the underneath wall to the topography boundary, which enables the microroller locomotion on the topography. However, depending on the microroller design and actuation frequency, the resistive forces can hinder microroller locomotion near the topographies. In order to assess the extent of the resistive forces caused by the nearby microtopographies, we performed 2D CFD analyses in which microrollers with fixed positions were rotated at different frequencies and gradually approached to the topography. For a concise comparison, we modeled the steepest structure (*h* = 6 µm, *c*_1_ = 300) as a 2D rectangle and single and doublet microrollers as an 8 µm circle and two conjoint 4 µm circles, respectively (Fig. 4*C*). For the same distance from the structure, flow field strength generated by the single microroller at 180 Hz was greater than 10 Hz actuation and the doublet microroller at 180 Hz (Fig. 4*D*). Rotational motion of the microrollers caused a fluidic pressure near the topography, which was more prominent for the single microroller compared to the doublet microroller when averaged over a complete rotation cycle (Fig. 4*D* and *SI Appendix*, Fig. S4). For all microroller groups, the total forces acting on the microrollers increased with decreasing the distance from wall (*d*); at distances close to the microtopography, the total forces in both horizontal and vertical directions experienced by the single microroller at 180 Hz were greater than both the single microroller at 10 Hz and the doublet microroller at 10 and 180 Hz (Fig. 4 *E* and *F*). The greater resistive forces acting on the single microroller in the simulations are in agreement with the experimental observations of their failure to propel over the structures. Of note, these simulations merely deal with microroller locomotion near the topography and do not concern climbing mode. Therefore, resistive forces in *y* axis are detrimental for locomotion and not useful for a microroller to locomote over the topography, unless the microroller has reached a certain distance from the topography boundary. To understand the nature of resistive forces caused by the topography, we decomposed the total force into viscous (*F*_*v*_) and pressure (*F*_*p*_) forces. For both microrollers rotating with 180 Hz at varying distances, the viscous force did not change considerably, while the pressure force increased greatly for the single microroller compared to the doublet microroller for both *x* and *y* axes. (Fig. 4*D* and *SI Appendix*, Fig. S5).

We ascribe the greater repulsive force experienced by the single microroller to the larger flow field area generated. To systematically investigate the generated flow fields around the single and doublet microrollers, we experimentally validated the generation of a larger flow vortex by the single microroller compared to the doublet when rotated in plane at different frequencies (10, 100, and 180 Hz) (*SI Appendix*, Fig. S6 *A* and *B*). Furthermore, we computationally modeled in-plane rotation of objects ranging from a perfect circle (isotropic) to ellipses (anisotropic) having the same length along with the doublet microroller. Even though all objects possess the same length, the isotropic shape generated the largest flow field magnitude (*SI Appendix*, Fig. S6*C*).

Rotational motion of the microrollers results in the pumping of fluid to their rotational direction. When placed near a solid microstructure (e.g., microtopography), pumped fluid between the microstructure and the microroller exerts additional resistive force due to incompressibility of the fluid. Flow field magnitude generated by the microrollers directly correlates with such extra resistive forces (Fig. 4 *E* and *F* and *SI Appendix*, Fig. S6, 10 and 180 Hz). Indeed, when a single microroller placed near a microtopography is simulated, resistive forces experienced by the microroller was much greater at 180 Hz compared to 10 Hz (Fig. 4 *E* and *F*), which is in agreement with the larger flow field generation at higher frequencies (*SI Appendix*, Fig. S6 *A* and *B*). Overall, the larger flow magnitude generated by the single microroller at 180 Hz near the structure results in larger repulsive forces, which prevents their translation closer to the microstructures. In accordance, the doublet microroller generates smaller flow field magnitudes; thus, experiences smaller repulsive forces, allowing it to propel closer to and over the microstructures (Fig. 4 *E* and *F*).

Other than microtopographies, flow fields generated by microrollers also interact with the narrow-channel boundaries, which could create additional resistive forces on the microrollers. A counterclockwise rotating microroller at a fixed position always experiences forces in negative direction without hydrodynamic confinement effects (32, 36). However, when the channel is confined beyond a certain threshold, magnitude of this force could decrease and eventually reverse in direction according to numerical simulations (36). We also observed the confinement effect in our simulations, in which a single microroller experienced positive total force, despite rotating counterclockwise in a 75 µm high microchannel. On the other hand, the doublet microroller experienced negative total force when actuated counterclockwise in the same channel dimensions. To systematically study the confinement effect, we performed additional simulations with different channel heights (50 to 500 µm), in which the single and doublet microrollers and anisotropic ellipses (*a*/*b* = 1.6, 5) were rotated at 180 Hz on a nearby solid wall (*SI Appendix*, Fig. S7*A*). The force experienced by a single microroller changed its direction (from negative to positive) after the channel height decreased below ∼150 µm, while the force direction of doublet microroller and ellipses did not change (*SI Appendix*, Fig. S7*B*). Furthermore, the total change in force magnitude between 50 and 500 µm channel heights for the single microroller was much larger than the other groups, indicating that the single microroller was affected the most from the confinement effect (*SI Appendix*, Fig. S7*C*). We also experimentally confirmed the existence of the confinement effect, in which the translational velocity of a single microroller decreased more than a doublet microroller under same confinement conditions (20 to 50 µm channel heights) (*SI Appendix*, Fig. S8). While we did not observe reversing motion in the experiments, the deviation between simulations and experiments could be ascribed to boundary conditions applied in the simulations (37) (for details, check *SI Appendix*, Note S2). While our simulations did not provide the exact solution for 3D microrollers and overestimated confinement effects, they provided a reasonable agreement for the force change trends for different microroller designs near microtopographical structures and under flow conditions, which were the main emphasis of this study.

### Propulsion of Microrollers on Microtopographies in Physiological Fluid Flows.

**Fig. 5.**
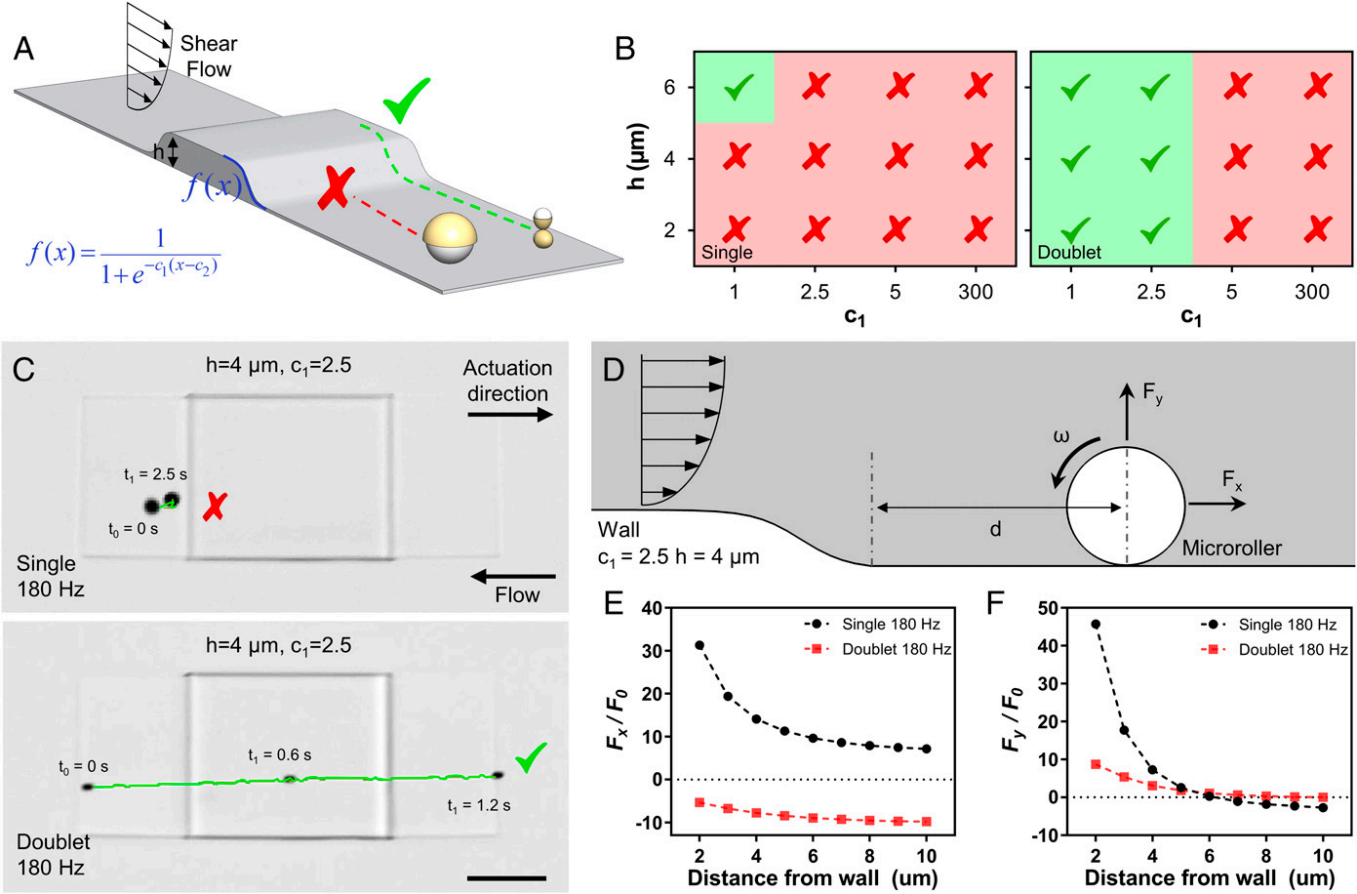
Upstream propulsion of microrollers on microtopographical surfaces under physiological flow. (*A*) The microrollers are driven upstream toward and over microfabricated topographical structures with controlled slopes and heights. (*B*) Performance diagrams of microrollers actuated at 180 Hz against physiological fluid flow on microtopographies. The single microrollers failed on almost all the structures, whereas the doublet microrollers could propel over structures with milder slopes (*c*_1_ = 1 to 2.5). (*C*) Time-lapse images of upstream propulsion of microrollers on microfabricated structures (*c*_1_ = 2.5, *h* = 4 µm). The single microroller failed, whereas the doublet microroller smoothly translated over the structure. (Scale bar, 50 µm.) (*D*–*F*) CFD analyses of microrollers near topographical structures. (*D*) A microroller with a fixed position in physiologically relevant fluid flow (1 dyn/cm^2^) was gradually approached to a structure (*c*_1_ = 2.5, *h* = 4 µm), and forces acting on the rollers were calculated. (*E*) Horizontal forces acting on the single microroller are in the direction of fluid flow and increased ∼fourfold when approached to the structure. On the other hand, the doublet microroller design experienced horizontal forces against the fluid flow direction, even when placed closest to the structure. (*F*) Vertical forces acting on the single microroller are greater than doublet microllers at close wall distances (*c*_1_ = 2.5, *h* = 4 µm, *d* = 2 to 5 µm).

Next, we tested the performance of both microroller types (at 180 Hz) against the physiologically relevant flow conditions (1 dyn/cm^2^ wall shear stress) on vessel-like microtopographical structures (Fig. 5*A*). The single microroller failed to locomote over almost all structures (11 out of 12), while the doublet microroller was successful with milder slopes (*c*_1_ = 1, 2.5) at all structure heights (Fig. 5*B*). For example, the single microroller failed to locomote over the structure with *h* = 4 µm and *c*_1_ = 2.5, whereas the doublet microroller was able to translate smoothly (Fig. 5*C* and Movie S2). The CFD analyses showed that the total resistive forces experienced by the single microroller are higher than the doublet, and the direction of the forces on *x* axis again was different because of the channel confinements (Fig. 5 *D*–*F*). The single microroller was only able to climb the structure with the mildest slope (*c*_1_ = 1) but only at the highest structure height (*h* = 6 µm). When we simulated forces on the single microroller around the same structure (*c*_1_ = 1) with varying heights (*h* = 2 to 6 µm), forces acting on the microroller near the highest structure were much smaller than the shorter structures (*SI Appendix*, Fig. S9), which explains the experimental observations.

### Microroller Propulsion on Vessel-like Synthetic and Biological Topographies in Physiological Fluid Flows.

**Fig. 6.**
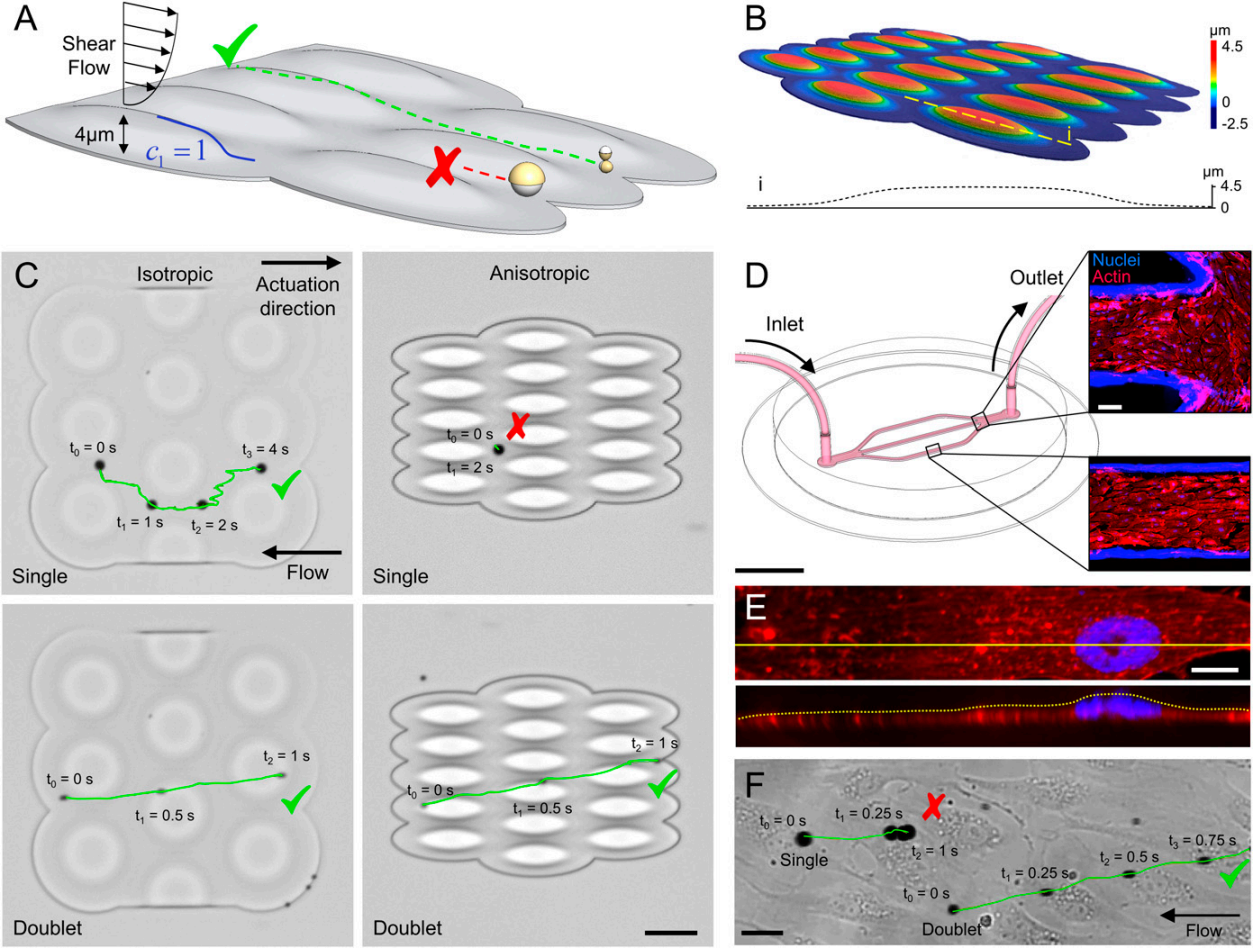
Upstream propulsion of microrollers on vessel-like microtopographies under physiological flow. (*A*) Microrollers (at 180 Hz) are driven upstream in physiological flow on structures mimicking endothelium (*c*_1_ = 1, *h* = 4 µm). (*B*) Laser confocal microscopy analysis of anisotropic vessel-like topographical microstructures. The *Inset* shows the height profile of the dashed line. (*C*) Time-lapse microscopy images of microroller propulsion on vessel-like topographies with isotropic (circular) and anisotropic (elongated) structures against physiological flow (1 dyn/cm^2^). The single microroller was able to propel on the isotropic topographies by following valleys formed in between but failed on anisotropic topographies. The doublet microrollers smoothly propelled over both structures. (Scale bar is 50 µm.) (*D*–*F*) Upstream propulsion of microrollers in endothelialized microfluidic channels. (*D*) The endothelialized microfluidic system with branched channels. (Scale bar, 5 mm.) The *Insets* show fluorescent microscopy images showing nuclei and actin fibers of endothelial cells at different sections of the microfluidic system. (Scale bar, 100 µm.) (*E*) Confocal microscopy image of a typical endothelial cell with a cross-sectional reconstruction of the z-stack planes. (Scale bar, 10 µm.) (*F*) Upstream propulsion of single and doublet microrollers on endothelial cells. Single microrollers failed to translate over endothelial cells, whereas doublet microrollers smoothly translated over cells. (Scale bar, 25 µm.)

Other than the morphology of individual cells, surface topography of the endothelium due to organization of the endothelial cells is also crucial for the motion of microrollers. In blood vessels, endothelial cells align and elongate in the direction of blood flow. To test the microroller propulsion in more realistic conditions, we fabricated vessel-like topographies, with structure heights around the same size of individual endothelial cells (∼4 µm), having isotropic (*c*_1_ = 1 in all directions) and anisotropic (*c*_1_ = 1 only in flow axis) slopes (Fig. 6 *A* and *B*). While the single microrollers were not able to propel over the isotropic structures, they were able to translate in the valleys formed in between the structures (Fig. 6*C* and Movie S3), which coincides with our previous in vitro isotropic microroller experimental observations on endothelial layers (9). On the other hand, the single microrollers were not able to propel at all over the anisotropic structures (Fig. 6*C*). The doublet microrollers, however, were able to locomote over both isotropic and anisotropic 3D topographies.

To investigate the microroller propulsion on real vessel-like topographies, we built a microfluidic vessel-on-a-chip system (Fig. 6*D*). After culturing endothelial cells in the microchannels under directional flows, the cells fully covered the microchannel surface, aligned and elongated in the flow direction, similar to the organization found in blood vessels (38) (Fig. 6*D*). The typical peak height of a single endothelial cell was around 2 to 5 µm, in accordance with the literature (11, 12), which mainly stems from the cell nucleus (Fig. 6*E*). When introduced into the endothelialized microchannels under physiologically relevant flow conditions (1 dyn/cm^2^), the single microrollers actuated at 180 Hz were able to partially locomote on some regions but stuck around the cell nuclei without any net translation (Fig. 6*F* and Movie S3). On the other hand, the doublet microrollers were able to locomote on the endothelial monolayer against the physiological flow.

## Discussion

The circulatory system consists of a closed network of vessels that carries oxygen and nutrients to all organs and the deepest tissues in the body. To overcome the harsh fluidic conditions in the blood vessels, the leukocytes in the body can roll on the vessels by taking advantage of low shear stresses and the cell-free layer on the walls (39, 40). Therefore, propulsion on the vessel walls, such as surface rolling or crawling, arises as the most viable approach for microrobot navigation in blood flow. While moving on the vessel wall is advantageous, surface topography is a crucial feature determining the surface motion capability, efficiency, and robustness of microrobots against the flow.

Microtopography of the blood vessels walls are complex in terms of the shape and orientation and can vary in different vessel types (41). Endothelial cells in blood vessels with higher-wall shear stresses (e.g., arterioles) are more elongated, whereas lower-wall shear stresses in other vessel types (e.g., postcapillary venules) results in lower aspect ratio (41). In addition to the topography of individual cells, the arrangement of endothelial cells on vessel walls is also a complex phenomenon, in which the shear stress distribution (42), blood flow continuity (16, 38), and biochemical factors (43, 44) are the main contributors. Two-photon lithography-based 3D microprinting allowed us to fabricate simplified biomimetic topographical structures with controlled dimensions and to systematically investigate the isotropic and anisotropic microroller propulsion. Experimental observations of microroller propulsion on 3D-printed microtopographies are also correlated with the vessel-on-a-chip model.

Microrobot shape is the determinant factor on the surface locomotion over topographical surfaces in a given fixed length scale. Therefore, we tested two different microrobot designs: the single and doublet microrollers, with different shape anisotropies. While their speed on flat surfaces was independent of their shape anisotropy, given that they have similar height, their propulsion on microtopographical structures was dramatically different with the doublet (anisotropic) microrollers outperforming the single (isotropic) microrollers. The CFD analyses and experimental results showed that the shape anisotropy determines the flow field generated around the microroller (*SI Appendix*, Fig. S6), and isotropic shapes, such as the single microroller, generate larger flow fields, resulting in higher repulsive forces from the environment. Therefore, rolling microrobots with high aspect ratios (height/diameter), such as microrods or microellipses, would be the most efficient, within the feasible micro/nanofabrication and physiological size-scale (i.e., the vessel diameter) limits. Such microrobots could be fabricated using both conventional (e.g., photolithography) and 3D (e.g., two-photon polymerization) microprinting techniques in a robust and standardized manner. Moreover, with decreasing vessel or channel size, the flow field generated around the microroller starts to interact with not only the nearby topographies but also the surrounding walls and creates extra forces on the microrollers against their translation direction. Therefore, rolling microrobots with slender bodies would be favorable for locomotion in smaller blood vessels.

In the body, wall shear stress values differ according to the vessel type, ranging from 0.76 to 7.6 dyn/cm^2^ in veins to 11.4 to 30.4 dyn/cm^2^ in large arteries (29). The wall shear stress value used in our study (1 dyn/cm^2^) corresponds to the values found in postcapillary and small venules in the body (29–31). Along with the topographical hurdles, a microroller should also overcome larger fluidic forces found in other blood vessels, which could be achieved by applying higher frequencies, resulting in higher translational speeds. For higher frequency actuation, magnetic properties of microrollers should be also high enough to prevent step-out under strong fluidic forces. Imaging of the microrollers in the body is another important consideration, though spatiotemporal resolution of the current imaging modalities used in the clinics are not high enough to image individual microrobots (∼8 µm). Swarm manipulation of a large number of microrollers could be beneficial in enhancing medical imaging contrast, and thus enable facile tracking and feedback control for the microrollers (45, 46). However, aggregation of microrobots, especially the ones with higher magnetic remanence, is a potential problem for swarm manipulation. This issue could be addressed by coating the microrobots with a nonmagnetic thick shell, such as silica, so that they can be disassembled using remote magnetic actuation methods if they aggregate.

Overall, we investigated the rolling performance of isotropic (sphere) and anisotropic (doublet spheres) microrollers against the physiologically relevant blood vessel-like conditions. Higher flow fields generated around the single microrollers impeded their motion near the microtopographies, while resistive forces were smaller for the doublet microroller. The CFD analyses indicated favorable hydrodynamic interactions for propulsion over topographical surfaces for the microrollers with higher shape anisotropy. Thus, our results suggest slender microroller designs, such as microrods, would provide the most efficient and robust rolling performance in physiologically relevant flow conditions.

## Materials and Methods

### Fabrication of Magnetic Microrollers.

Magnetic Janus microrollers were fabricated by sequentially sputtering 1 μm thick Ni and 20 nm thick Au nanofilm layers on predried monolayers of silica particles (3 or 8 μm diameters, SiO_2_, microParticles GmbH) using a sputter coating system with predefined tilt angles (Leica EM ACE600, Leica Microsystems). Magnetization direction of the Janus microparticles was aligned to an out-of-plane direction by applying 1.8 T uniform magnetic field inside a VSM (MicroSense). The sputtered and magnetically aligned microparticles were released from the substrate using a bath sonicator in ethanol, washed several times, and then dispersed in phosphate buffered saline (PBS) 1×. Doublet microrollers were formed by magnetic self-assembly of 3 μm diameter Janus particles. Briefly, 350,000 particles/mL of 3 μm diameter Janus particle solution in a vial was sonicated and then vortexed for 5 min. During this process, particles with different sizes and structures were formed (e.g., doublet, triplet, etc.), and only doublet microrollers were used in the experiments.

### Magnetic Field Control.

The microrollers were actuated using a custom-built five-coiled electromagnetic coil system (4 *xy* coils and 1 *z* coil) placed on an inverted microscope (Zeiss Axio Observer A1, Carl Zeiss). Specifically, out-of-plane 10 mT rotational magnetic fields were applied to the microrollers, which resulted in surface-enabled locomotion. The rolling direction of the microrollers was controlled by changing the orientation of the out-of-plane magnetic field.

### Actively Controlled Locomotion in Upstream Flow.

Upstream flow experiments were performed in a closed microchannel composed of a poly(methyl methacrylate) (PMMA) top, a double-sided tape, and a glass slide. The PMMA top piece encloses the fluidic connections, the double-sided tape defines the channel shape and height, and the glass slide makes the basement (47). PMMA and double-sided tape were micromachined using a CO_2_ laser cutting system (Epilog Laser) to achieve a microchannel having the dimensions of 75 μm height by 3 mm width by 12 mm length. The microrollers were mixed with PBS 1×, loaded into a syringe, and pumped into the microfluidic channels at a controlled flow rate. The flow rate was adjusted to generate the desired wall shear stress value (1 dyn/cm^2^), which was calculated according to the following:

$$\tau =\frac{6\eta Q}{wh^{2}},$$ [5]

where *h* and *w* are the channel height and width, respectively, *η* is the dynamic fluid viscosity, and *Q* is the volumetric flow rate.

### 3D Microprinting of Topographical Structures.

The topographical structures were fabricated from IP-S photoresist using a commercially available two-photon lithography system (Photonic Professional, Nanoscribe GmbH). The slopes of the obstacles were fabricated using the basic Sigmoid function

$$f\left(x\right)=\frac{1}{1+e^{-c_{1}\left(x-c_{2}\right)}},$$ [6]

where *c*_1_ and *c*_2_ determine the slope and midpoint on *x* axis, respectively. The 3D computer-aided design files were generated using *c*_1_ = 1, 2.5, 5, and 300 and *c*_2_ = 0.5 for all files in SolidWorks (Dassault Systèmes). To adjust the height of the obstacles, the structures were scaled up to a factor of 2, 4, and 6 (2, 4, and 6 μm in height). All printings contain 1 µm thick basement layer to ensure the quality of the printing. Microtopographical structures used in static experiments were fabricated with an 80 μm width. All the structures used in flow experiments were fabricated with 140 μm length and 100 μm width. In vessel-like synthetic topographies, *h* = 4 μm and *c*_1_ = 1 structures were used as the template. Circular structures were created by rotating the function around the *y* axis and aligned in a hexagonal order. Elliptic structures were created by anisotropically compressing the circular structures from one side to mimic aligned endothelial monolayers. For the confinement effect experiments, 500 ×500 ×10, 20, or 30 μm blocks were microprinted on the top of a 50 μm height microchannel. SEM imaging of the 3D-printed structures was performed via a ZeissUltra 550 Gemini scanning electron microscope (Carl Zeiss Inc.). Height and profile of the fabricated microstructures and channels were further measured with an optical profilometer (VK-X250, Keyence).

### Cell Culture Experiments.

For branched endothelialized microfluidic channels, double-sided adhesive tape was micromachined using an ultraviolet laser system (LPKF ProtoLaser U3) with a width of 300 µm for the smallest channel. Human umbilical vein endothelial (HUVEC) cells were grown in endothelial cell growth basal medium 2 (CC-3156, Lonza) supplemented with endothelial cell growth media 2 SingleQuots (CC-4176, Lonza) in a 5% CO_2_, 95% air humidified atmosphere. After reaching to confluence, HUVEC cells were trypsinized and then introduced into the microchannels coated with fibronectin (0.1 mg/mL for 1 h at room temperature) at a concentration of 10^7^ cell/mL and cultured with media flow at 4 dyn/cm^2^ for 2 d.

### Computational Fluid Dynamics (CFD) Simulations.

COMSOL Multiphysics 5.5 Simulation Software (COMSOL, Inc.) was used to estimate flow fields, pressure distribution, and the forces acting on the microrollers in static and flow conditions. Simulations were performed in a 2D plane, by solving the Navier–Stokes equations for single and doublet microrollers placed in a rectangular microfluidic channel filled with deionized water as media. Top and bottom planes of the channel were defined as no-slip boundaries, and a separation distance of 200 nm was used between the microrollers and the bottom surface. Flow fields induced by the rollers were defined as tangential velocities at different actuation frequencies (10 to 180 Hz) with respect to their distance to the roller center. Using the creeping-flow interface physics, the steady-state forces and velocity fields were then obtained. The forces acting on the microrollers were calculated using the following:

$$F=\overset{\;}{\underset{\partial \Omega }{\int }}\sigma \cdot n\;dS,$$ [7]

where *σ* is the stress tensor, ∂Ω is the microroller surface boundary in 2D, and *n* is the outward pointing normal vector. The average 2D forces (total force per unit depth of microrollers) were calculated by taking the average of the forces over one cycle. All the forces in the study were normalized to *F*_*0*_ = 10^−6^ N/m for easier comparison.

### Particle Tracing Measurements.

The images were taken using a high-speed camera (M310; Phantom, Inc.) with 1,000 frames per second. The images were then analyzed using a Trackpy Python package to find the trajectories of 1 μm tracer particles around the rotating microrollers.

### Statistical Analysis.

All quantitative values were presented as means ± SD of the mean.

## Supplementary Material

Supplementary File

Supplementary File

Supplementary File

Supplementary File

## Data Availability

All study data are included in the article and/or supporting information.
