# Supplementary material for: Shape anisotropy-governed locomotion of surface microrollers on vessel-like microtopographies against physiological flows
Source: Proc Natl Acad Sci U S A. 2021 Mar 22;118(13):e2022090118. doi: 10.1073/pnas.2022090118 (PMC8020797; doi:10.1073/pnas.2022090118)
Supplement: Supplementary File [file pnas.2022090118.sapp.pdf]

Supplementary Information

Shape anisotropy-governed locomotion of surface microrollers on vessel-like microtopographies against physiological flows

Ugur Bozuyuk[1, 2#], Yunus Alapan[1#], Amirreza Aghakhani[1], Muhammad Yunusa[1], Metin Sitti[1, 2, 3*]

[1] Physical Intelligence Department, Max Planck Institute for Intelligent Systems, 70569 Stuttgart, Germany

[2] Institute for Biomedical Engineering, ETH Zurich, 8092 Zurich, Switzerland

[3] School of Medicine and College of Engineering, Koç University, 34450 Istanbul, Turkey

* Correspondence to: sitti@is.mpg.de

[#] These authors contributed equally and share the first authorship.

Note S1.

While a microroller in motion, gravitational forces against buoyancy (F_G) are balanced out with electrostatic repulsion (F_{rep}) and lift forces (F_L) on *y*-axis (1-3):

$$F_g = \frac{4}{3}\pi(\rho_p - \rho_f)a^3 g \qquad [S1]$$

$$F_{rep} = 4\pi\varepsilon\varepsilon_0 a\kappa v_1 v_2 e^{-\kappa\delta} \qquad [S2]$$

$$F_L = \pi\rho_f a^3 \omega U \qquad [S3]$$

where $\varepsilon\varepsilon_0$ is the permittivity of the medium, κ^{-1} is Debye length, v_1 is the zeta potential of the particle, v_2 is the zeta potential of the near surface, ρ_f is the density of the fluid, ρ_p density of the particle, and g is the gravitational constant. However, F_L is negligible in the given size scale of the microroller in our study (<10 µm) (4). From the equilibrium on *y*-axis, separation distance is expressed as:

$$\delta = -\frac{1}{\kappa}\ln\left(\frac{\frac{4}{3}\pi(\rho_p - \rho_f)a^3 g}{4\pi\varepsilon\varepsilon_0 a\kappa v_1 v_2}\right) \qquad [S4]$$

Note S2.

The hydrodynamic forces acting on a fixed sphere rotating in counterclockwise direction should be in the left (negative) direction (Fig. 4a) on the *x*-axis without any confinement according to the lubrication theory (5, 6). However, in our simulations, forces on the single microroller rotated in the counterclockwise direction were in the right (positive) direction when modeled within a microchannel with a height of 75 µm and width of 500 µm. To understand the effect of confinement on hydrodynamic forces experienced by the microrollers, we modeled microchannels with varying heights (50 µm – 500 µm) (Fig. S7a). When rotated in the counterclockwise direction, the doublet microroller experienced forces in the left direction independent of the channel dimensions (Fig. S7b), whereas the single microroller experienced forces in the right direction until a certain channel dimension (~150 µm). Above these dimensions, the forces acting on the single microroller were in the left direction and converged to a certain value (Fig. S7b). Note that the experiments were performed in a 75 µm-high channel, where counterclockwise rotation of the single microrollers resulted in movement in left direction for single microrollers in contrast to the simulation results. Our simulations and previous literature (6) have shown such confinement effects exist, however, we did not observe any reversing motion in the experiments. To experimentally investigate the confinement effects, we actuated the single and doublet microroller at 180 Hz in microchannels with 20, 30, 40 and 50 µm height (Fig. S8a,b). We observed decreases in translational velocity for both microrollers with decreasing channel height due to extra repulsion forces acting on the microrollers (Fig. S8c). However, decrease in translational speed, thus confinement effect, on the single microroller was more pronounced, especially in 20 µm height. A similar trend in terms of the repulsive forces was also obtained in the simulations with the exception of force direction change for the single microroller in channel heights smaller than ~150 µm (Fig. S7b). We ascribed this discrepancy to the no-slip boundary condition defined in the simulations (7). A recent study (6) on surface microrollers showed that the confinement effects with no-slip boundary condition causes a force and movement direction change (*i.e.*, counterclockwise rotation but moving in positive direction) in CFD simulations. However, when slip boundary condition is applied, a microroller continues to translate in its usual direction (*e.g.*, clockwise, positive direction) even in a very confined condition, but with slower translational speeds, as observed in our experimental findings. These findings indicate that the partial slip on the microchannel walls (7-9) (*i.e.*, partial wetting of channel surfaces), may have caused the deviations between our experiments and simulations. Other than walls of the microchannels, surface wettability of microrobots may have contributed to this effect as well (10). We would like to pay the readers' attention to the references (7, 11) for more details.

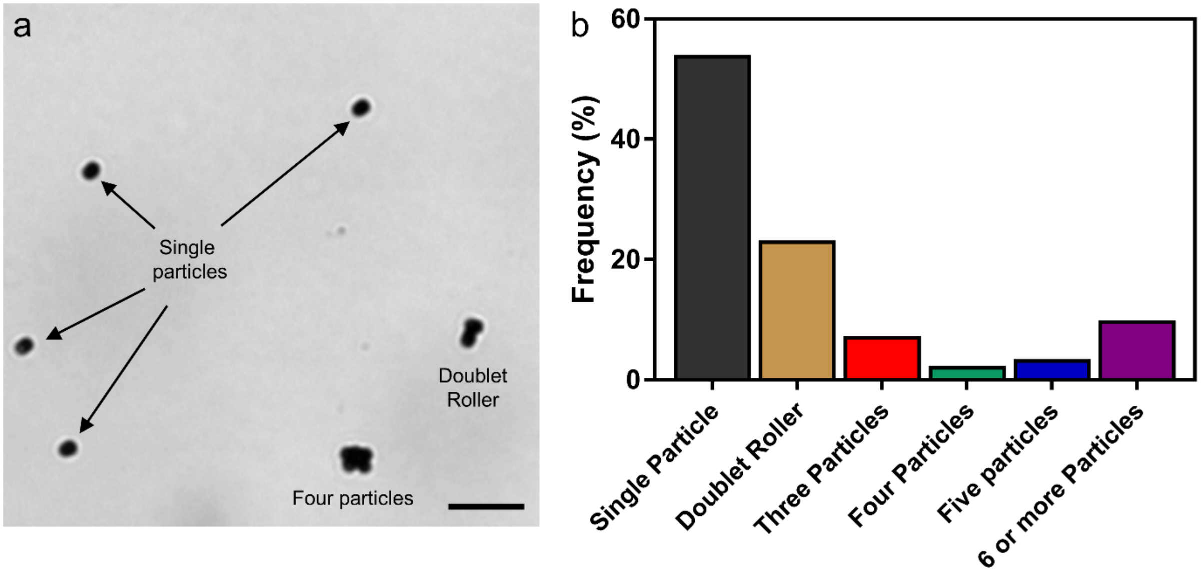

Figure S1. Frequency distribution of magnetically self-assembled 4 µm-diameter Janus particles. a) Light microscopy image of magnetic Janus particles showing the distribution of single particles and self-assembled particles. In doublet formation, one Janus particle assembles to another one from the side. **b)** Frequency distribution shows that more than 20% of the particle aggregates were doublet microrollers, whereas more than 50% were single particles.

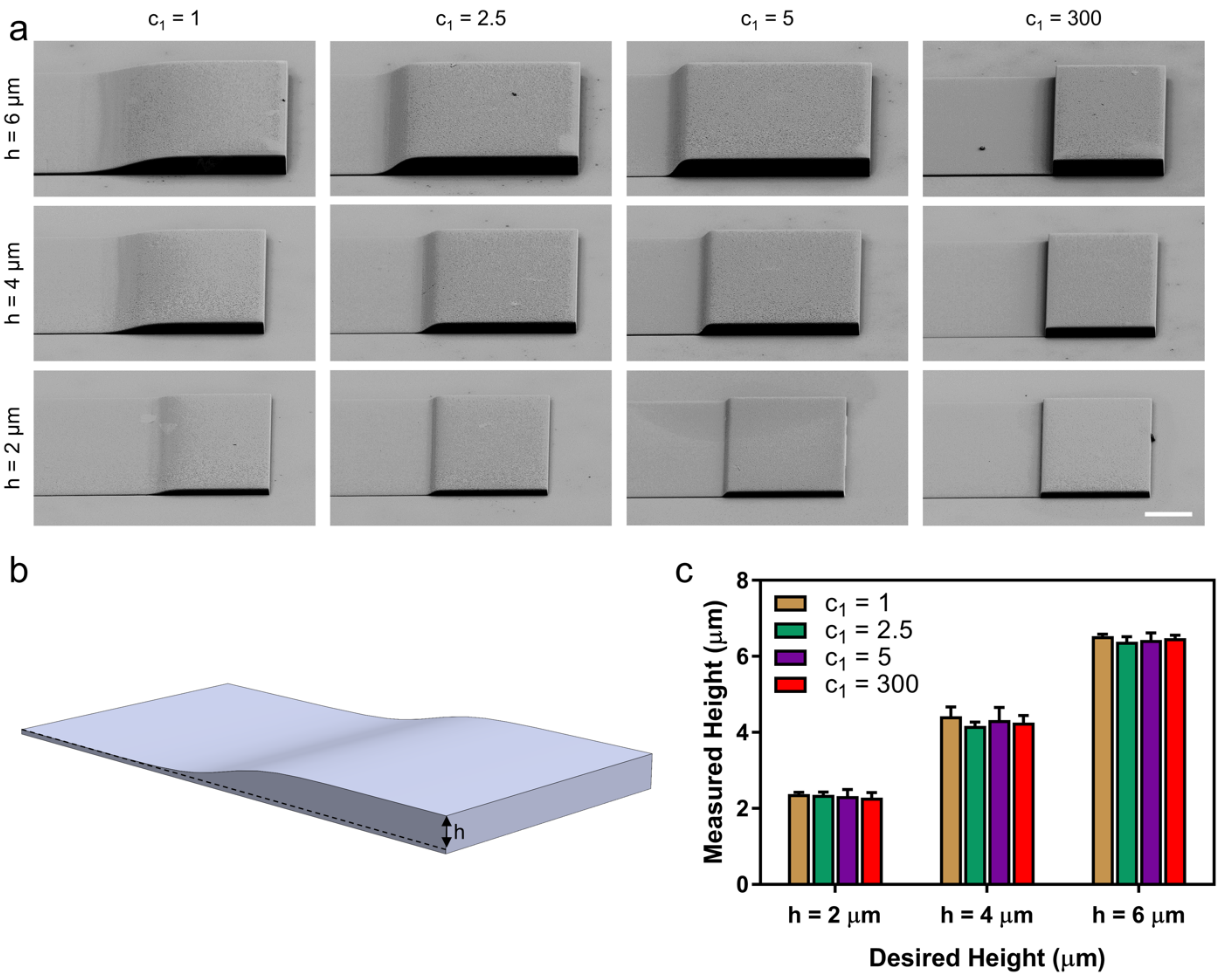

Figure S2. Characterization of microfabricated microtopographical structures. a) SEM images of fabricated microstructures with varying slopes (c_1 = 1-300) and heights (h = 2-6 µm). **b-c)** Comparison of desired and measured heights of the fabricated topographical structures analyzed with a laser confocal microscopy. The measured height of the structures is slightly higher than the desired heights (0.2 - 0.4 µm for all structures), where the differences are in the resolution limit of the used objective in microprinting. Error bars represent the standard deviation of the mean.

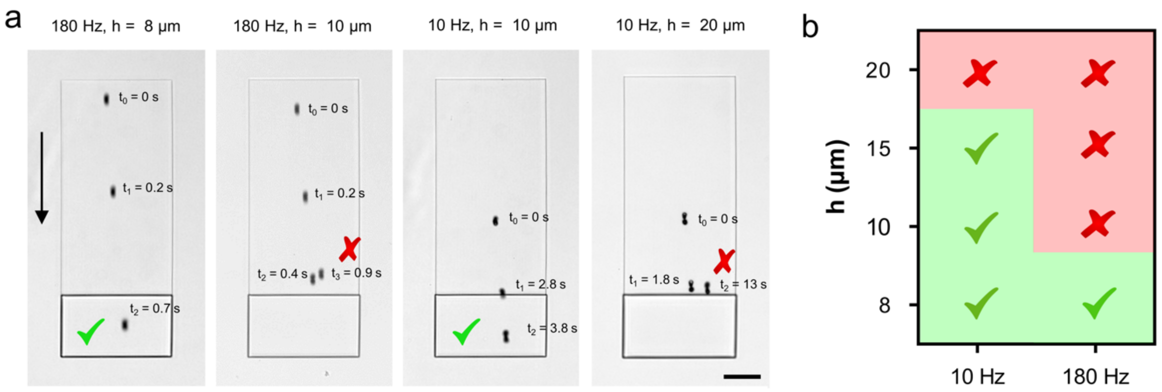

Figure S3. Propulsion of doublet microrollers over higher microstructures with c_1 = 300. a) Time-lapse images of the doublet microroller on microfabricated structures with different wall heights. The doublet microroller was able to locomote over the *h* = 8 μm wall at 180 Hz but failed at *h* = 10 μm at the same frequency. At 10 Hz, the doublet microroller was able to locomote over the *h* = 10 μm wall, but failed *h* = 20 μm wall. **b)** Performance diagram of the doublet microroller showing the limits of the doublet microroller with two different rotational frequencies (10 and 180 Hz).

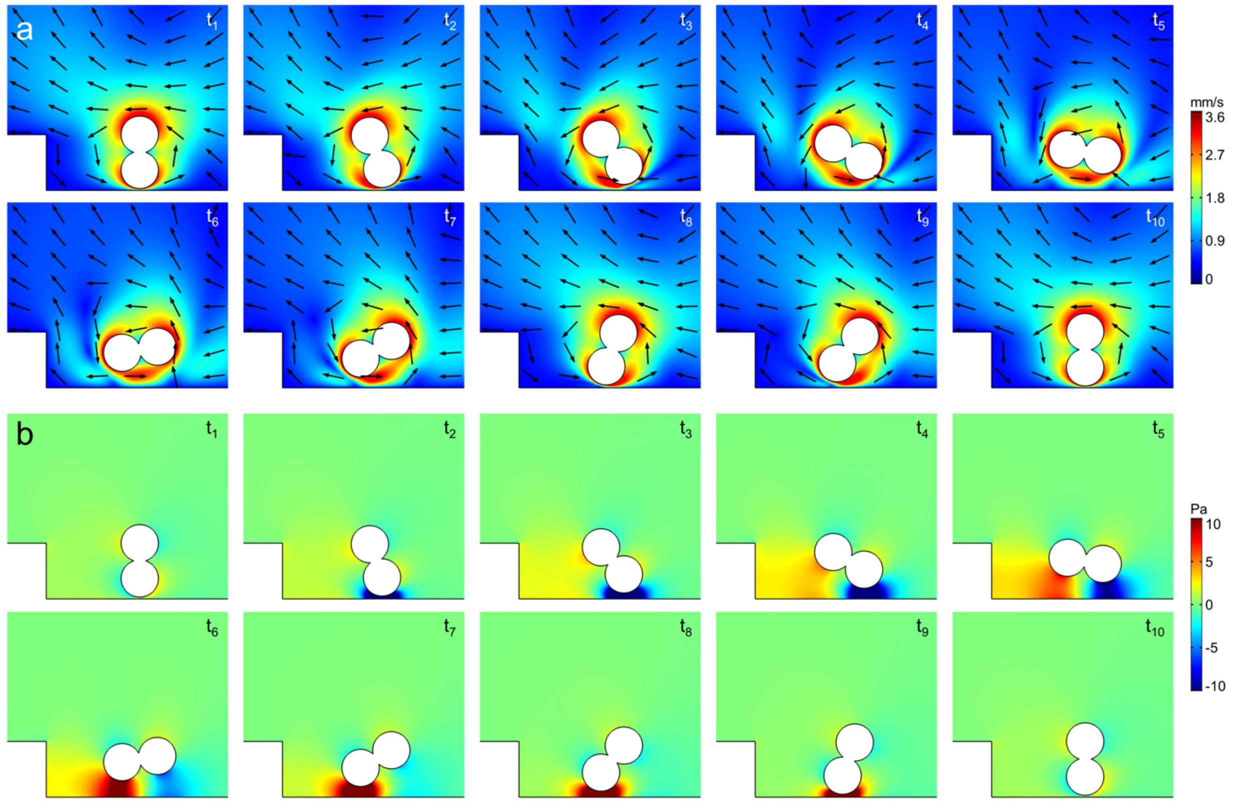

Figure S4. Computational fluid dynamics (CFD) analyses of flow field strength (a) and pressure distribution (b) for doublet microrollers in a rotation cycle. Black arrows indicate fluid flow direction.

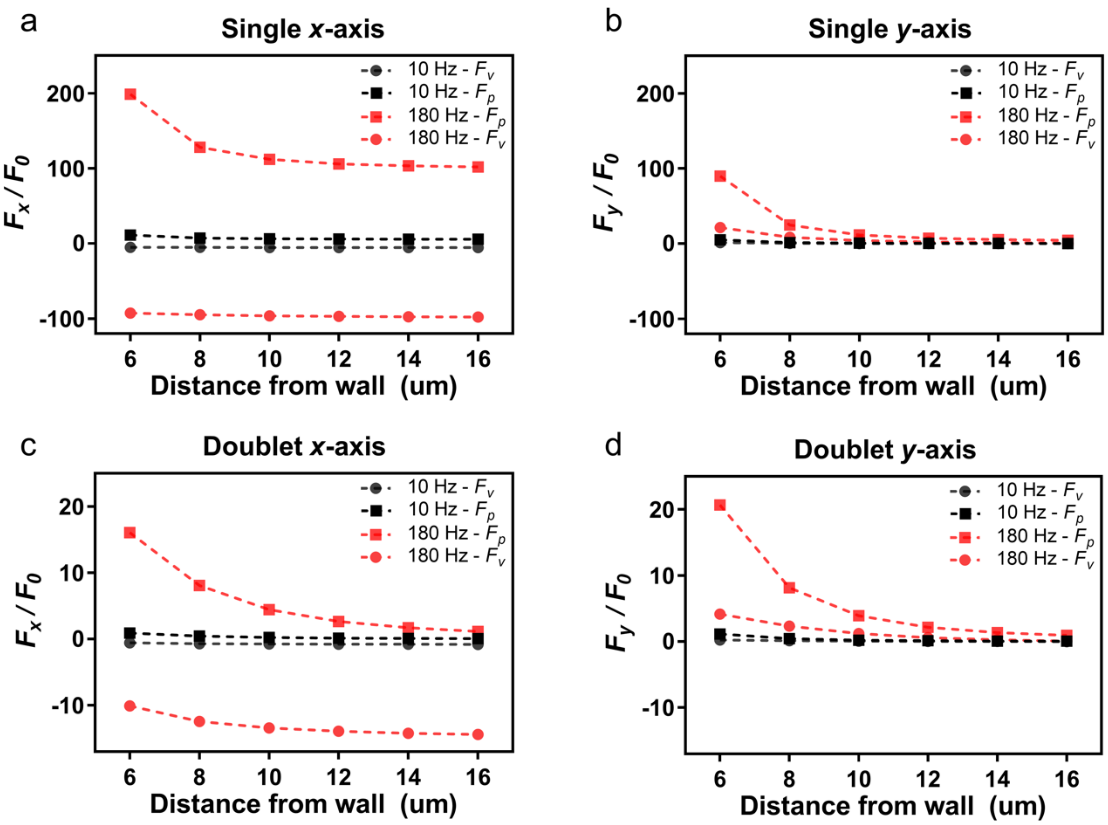

Figure S5. The viscous (F_v) and pressure forces (F_p) acting on the single and doublet microrollers at 10 and 180 Hz around a topographical structure (h = 6 µm, c_1 = 300). a-d) Viscous forces remain relatively stable for single and doublet microrollers, while pressure forces dramatically increase for single microrollers at closer wall distances on *x*-axis. Pressure forces acting on the single microroller on *y*-axis also increases greatly, compared to the doublet.

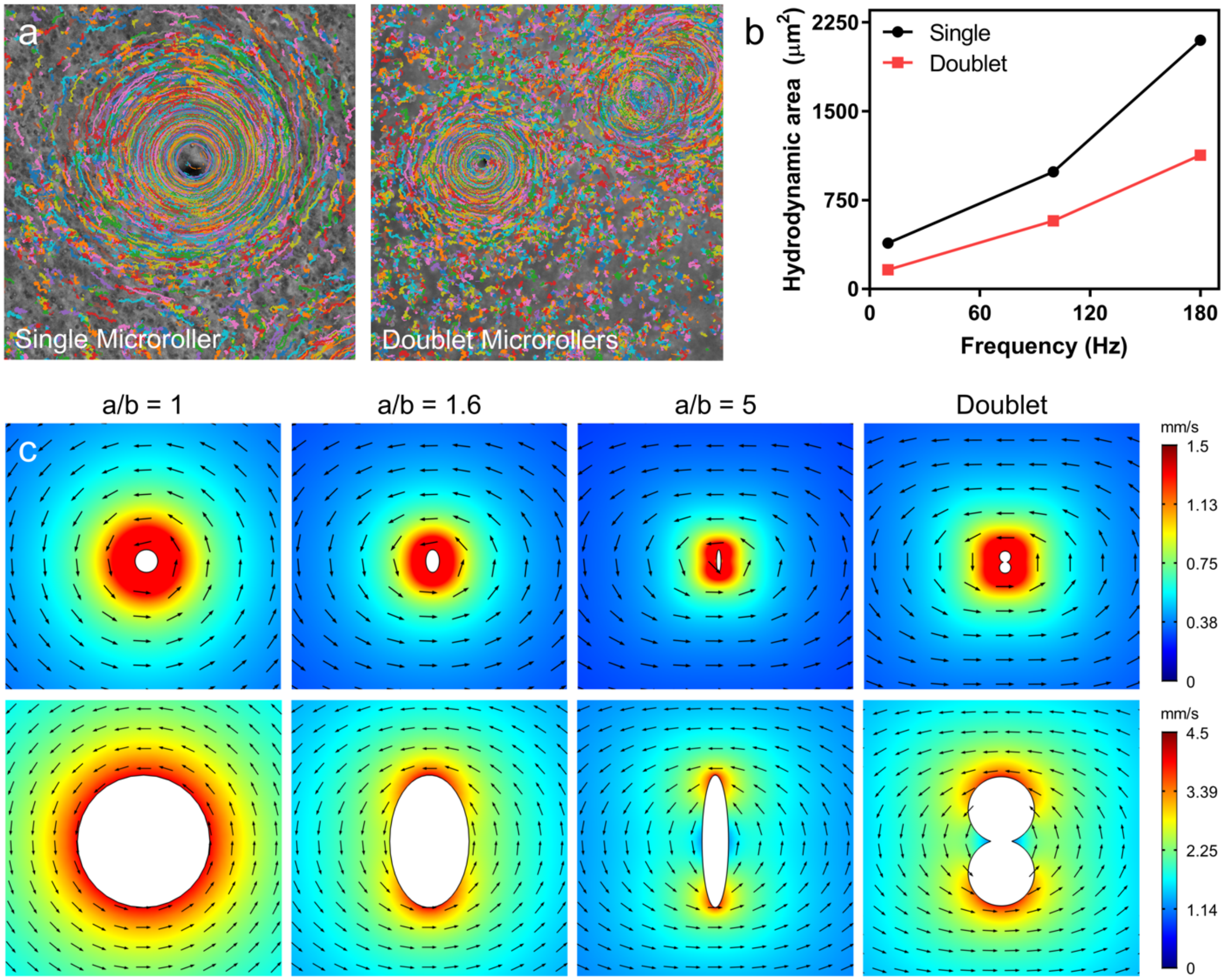

Figure S6. Hydrodynamic flow field around in-plane rotating microrollers with varying shape anisotropies. a) Particle image tracing analyses showing 1 µm particle trajectories for single and doublet microrollers actuated at 180 Hz in in-plane counterclockwise direction. **b)** Single microroller generated larger hydrodynamic area compared to doublet microrollers at all actuation frequencies. **c)** Computational fluid dynamics analyses of flow field strength for in-plane rotating microrollers with varying shape anisotropy (a/b = 1-5). The spherical shape (a/b = 1) generates a larger hydrodynamic flow field compared to anisotropic shapes, including doublet microroller (a/b = 1.6, 5). The bottom row shows close-up images of flow field generated around different shapes. Black arrows indicate fluid flow direction.

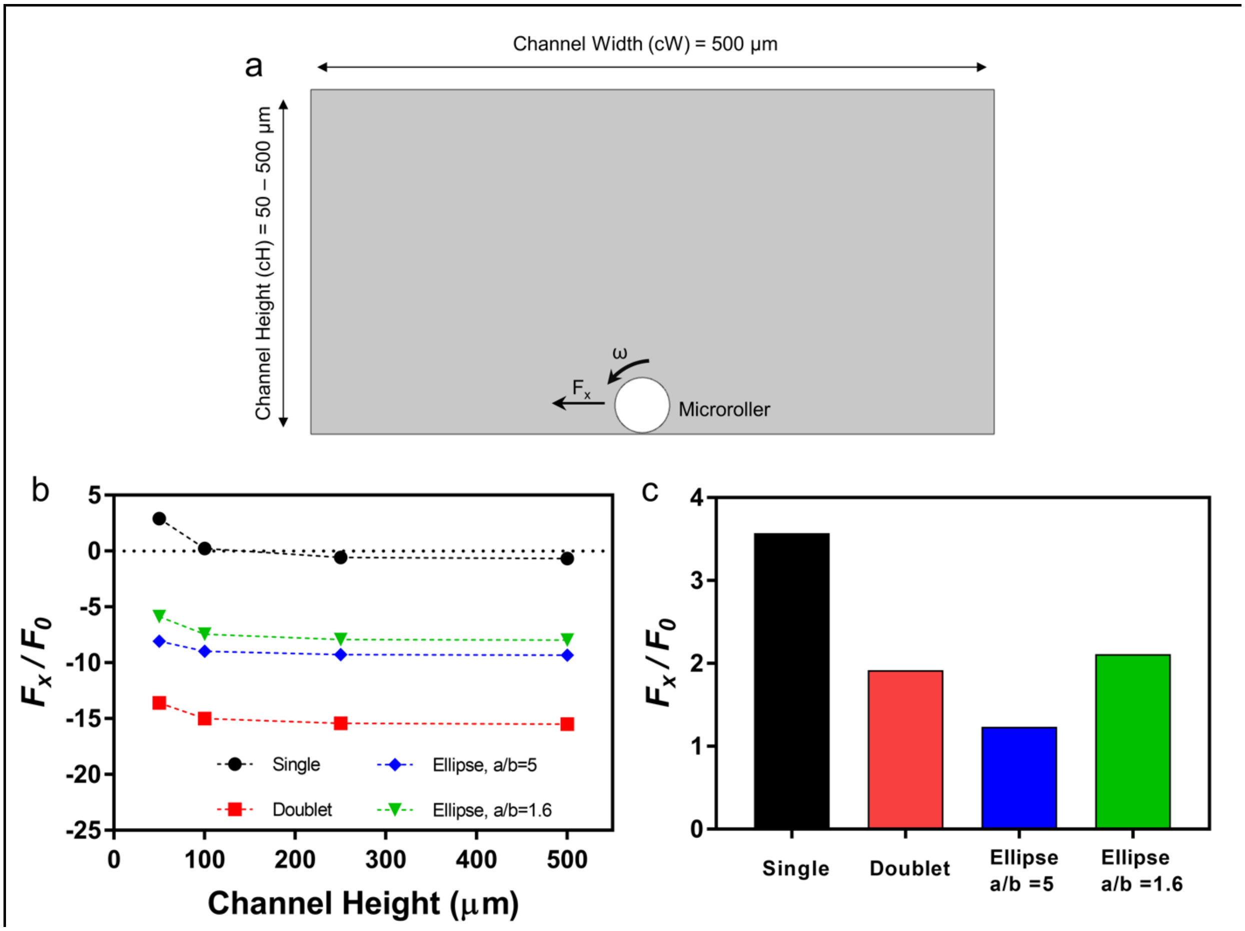

Figure S7. CFD analyses of forces acting on a microroller depending on the microchannel height. a) A microroller with a fixed position near the bottom wall was rotated in counterclockwise direction at 180 Hz for varying channel heights (50 – 500 µm). **b)** The single microroller starts to experience positive forces for channel heights smaller than ~150 µm. On the other hand, the forces acting on the doublet and the elliptic microrollers were negative irrespective of the channel height, though still decreased with decreasing height. **c)** Total force magnitude change for the different microroller groups between 50 and 500 µm channel heights, indicating the single microroller is affected the most by the channel confinement.

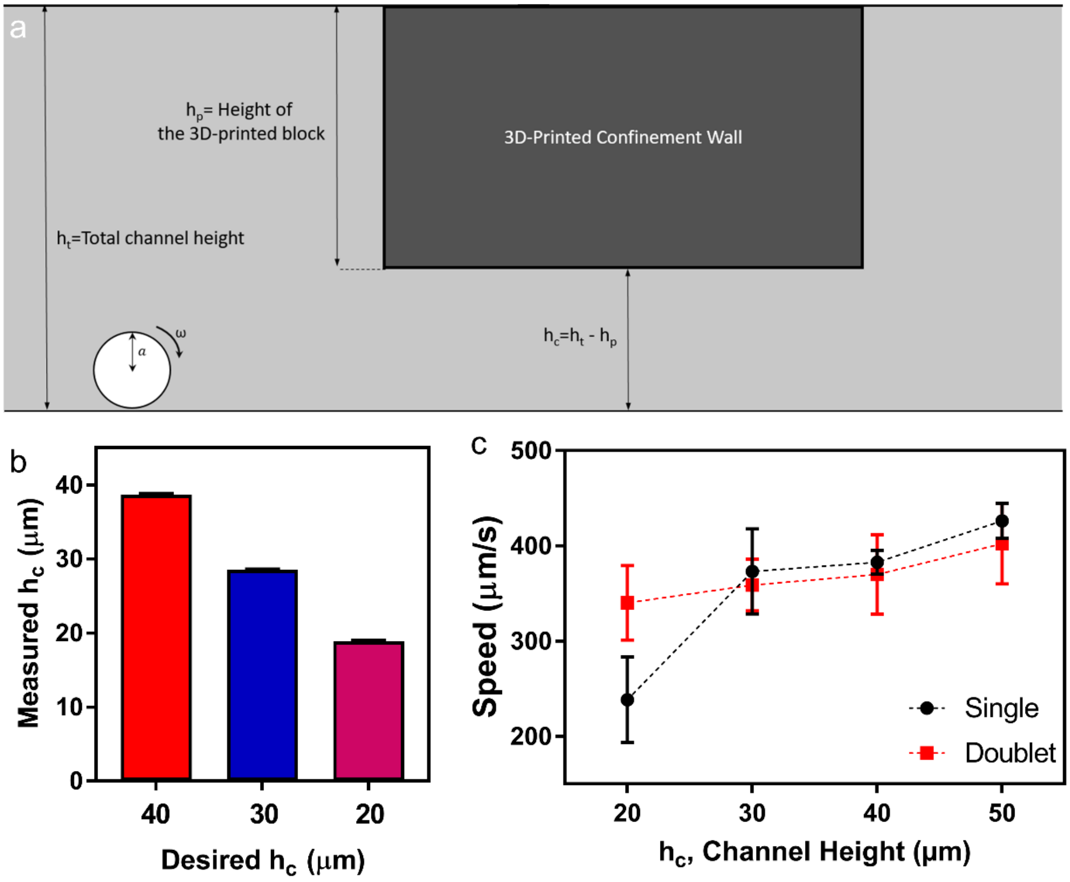

Figure S8. The experimental confinement effect on the single and the doublet microroller. a) A schematic showing the experimental configuration. Rectangular blocks with known height (h_p) were 3D microprinted to the top of the channels, and thus the height of the confined channels (h_c) were adjusted by substracting the total channel height (h_t) from the h_p. **b)** The desired versus the measured height of the confined channels in laser confocal microscopy. The channels were approximately ~1 µm smaller than the desired h_c. **c)** Average speed of the microrollers in different h_c. The average speed of the single microroller dropped dramatically at 20 µm channel, while the decrease in the doublet microroller was milder, showing the confinement effect was more severe for the single microroller.

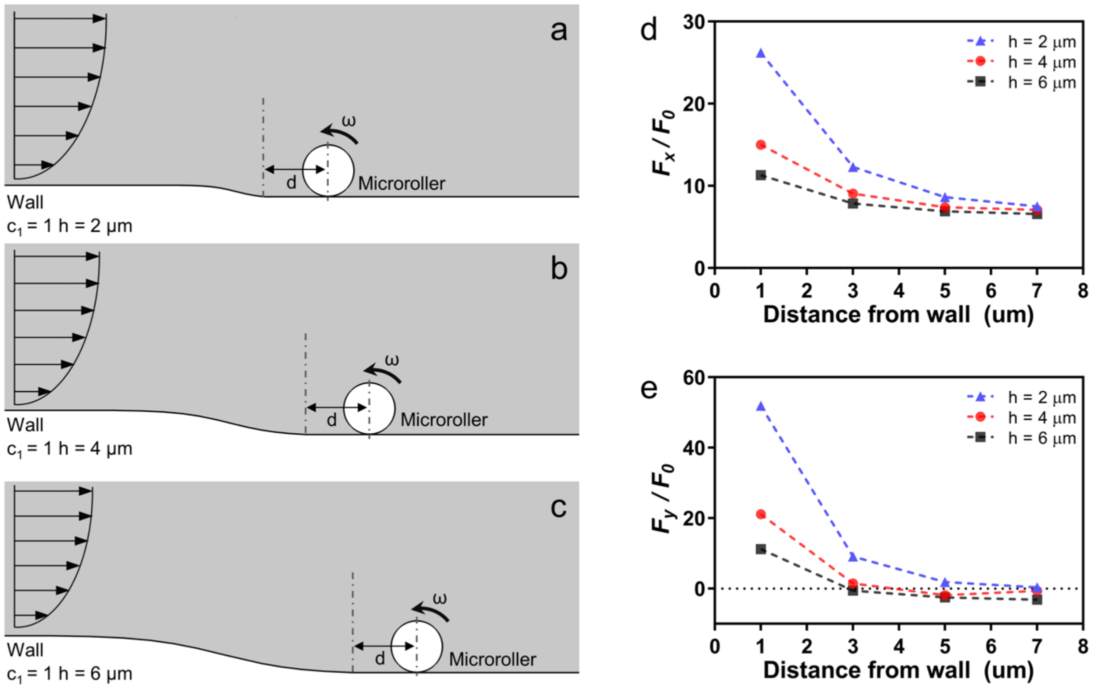

Figure S9. CFD analyses of single microrollers near the structures with the mildest slope and varying heights under physiological fluid flow. a-c) The single microrollers rotating against physiologically relevant fluid flow (1 dyn/cm^2) was gradually approached to structures with different heights (c_1 = 2.5, h = 2-6 µm) and forces acting on the rollers were calculated. Wall distances (d) were defined between the microroller center and the point where the slope ends. **d, e)** Forces acting on the single microrollers in the horizontal (x) and the vertical (y) direction depending on the wall distance. The forces acting on single microrollers increased with decreasing structure height and were largest when the structure height was 2 µm. All the forces were normalized to forces acting on the microrollers placed closest to the shortest structure (h = 2 µm, d = 1 µm).

References

1. T.-Y. Chiang, D. Velegol, Localized Electroosmosis (LEO) Induced by Spherical Colloidal Motors. *Langmuir* **30**, 2600-2607 (2014).
2. T. Li *et al.*, Janus Microdimer Surface Walkers Propelled by Oscillating Magnetic Fields. *Advanced Functional Materials* **28**, 1706066 (2018).
3. S. I. Rubinow, J. B. Keller, The transverse force on a spinning sphere moving in a viscous fluid. *Journal of Fluid Mechanics* **11**, 447-459 (1961).
4. A. Kaiser, A. Snezhko, I. S. Aranson, Flocking ferromagnetic colloids. *Science Advances* **3**, e1601469 (2017).
5. A. J. Goldman, R. G. Cox, H. Brenner, Slow viscous motion of a sphere parallel to a plane wall—I Motion through a quiescent fluid. *Chemical Engineering Science* **22**, 637-651 (1967).
6. W.-Z. Fang, S. Ham, R. Qiao, W.-Q. Tao, Magnetic Actuation of Surface Walkers: The Effects of Confinement and Inertia. *Langmuir* **36**, 7046-7055 (2020).
7. E. Lauga, M. Brenner, H. Stone, "Microfluidics: The No-Slip Boundary Condition" in Springer Handbook of Experimental Fluid Mechanics, C. Tropea, A. L. Yarin, J. F. Foss, Eds. (Springer Berlin Heidelberg, Berlin, Heidelberg, 2007), 10.1007/978-3-540-30299-5_19, pp. 1219-1240.
8. E. Lauga, M. P. Brenner, Dynamic mechanisms for apparent slip on hydrophobic surfaces. *Physical Review E* **70**, 026311 (2004).
9. E. Lauga, H. A. Stone, Effective slip in pressure-driven Stokes flow. *Journal of Fluid Mechanics* **489**, 55-77 (2003).
10. X. Wang *et al.*, Surface-Chemistry-Mediated Control of Individual Magnetic Helical Microswimmers in a Swarm. *ACS Nano* **12**, 6210-6217 (2018).
11. G. Karniadakis, A. Beskok, N. Aluru, *Microflows and nanoflows: fundamentals and simulation* (Springer Science & Business Media, 2006), vol. 29.

Supplementary Movies

Supplementary Movie 1. Magnetic actuation of single and doublet microroller on flat surfaces

Supplementary Movie 2. Characterization of microrollers on microtopographical surfaces

Supplementary Movie 3. Characterization of microrollers on vessel-like microtopographies against physiological flow
